# Polysaccharides-gut microbiota interaction: mechanisms regulating the hepatocellular carcinoma immune microenvironment

**DOI:** 10.3389/fimmu.2026.1789273

**Published:** 2026-06-08

**Authors:** Wei Peng, Kai Xiong, Yuyang Zheng, Jiahan Zheng, Yuanyuan Zhong, Jihao Yang, Yuchuan Jiang

**Affiliations:** 1 Department of Graduate School, The First Clinical Medical College of Gannan Medical University, Gannan Medical University, Ganzhou, China; 2 Department of Gastroenterology, The Second Affiliated Hospital, Jiangxi Medical College, Nanchang University, Nanchang, China; 3 Department of Pediatric Surgery, The First Clinical Medical College of Gannan Medical University, Ganzhou, China; 4 Department of Gastroenterology, Pingxiang People’s Hospital, Pingxiang, Jiangxi, China; 5 School of Acupuncture and Tuina, Guizhou University of Traditional Chinese Medicine, Guiyang, China

**Keywords:** Gut microbiota, gut-liver axis, hepatocellular carcinoma, natural polysaccharides and nanopolysaccharides, tumor immune microenvironment

## Abstract

Hepatocellular carcinoma (HCC) has a poor prognosis, and the clinical responses to immune checkpoint inhibitors (ICIs) remain limited. Increasing evidence suggests that gut microbiota dysbiosis plays an important role in HCC progression through the gut-liver axis. This review summarizes the mechanisms by which polysaccharide-gut microbiota interactions reshape the immunosuppressive tumor microenvironment (TME) in HCC, and discusses the translational potential and challenges of this emerging therapeutic axis. Specifically, gut microbiota dysbiosis promotes chronic hepatic inflammation and immunosuppression through metabolites such as lipopolysaccharide, short-chain fatty acids, and bile acids. As biocompatible prebiotics, natural polysaccharides can selectively enrich beneficial gut bacteria, including Bacteroides and Akkermansia, promote the production of immunoregulatory metabolites, and regulate key signaling pathways such as TLR/NF-κB, bile acid-FXR, and PD-1/PD-L1. Nanopolysaccharides designed to improve tumor-targeting efficiency are also being explored in preclinical studies for HCC. Despite the therapeutic potential of the gut microbiota-polysaccharide-liver TME axis, several challenges remain, including polysaccharide structural heterogeneity, unclear microbiota-immune causal relationships, and undefined safe dose windows. Overall, this review provides an integrated overview of polysaccharide-based modulation of the HCC immune microenvironment and may offer insights for the development of more precise therapeutic strategies according to HCC etiological heterogeneity.

## 1 Introduction

Hepatocellular carcinoma (HCC) accounts for approximately 90% of primary liver cancers and remains a major cause of cancer-related mortality worldwide (1–4). Although immune checkpoint inhibitors (ICIs) have expanded treatment options for HCC, their clinical benefits remain limited, and only 20–30% of patients achieve durable responses (5–8). Increasing evidence suggests that the immunosuppressive tumor microenvironment (TME) and gut microbiota dysbiosis are major contributors to ICI resistance (7, 8). This therapeutic challenge has prompted growing interest in the regulatory factors that shape the HCC immune microenvironment. Among them, the gut microbiota has emerged as a key regulator of hepatic immune homeostasis and tumor progression through the gut-liver axis. Microbial metabolites, including lipopolysaccharide (LPS), short-chain fatty acids (SCFAs), and bile acids (BAs), directly influence the hepatic TME (9–11). Under physiological conditions, the gut microbiota helps maintain host immune tolerance. In contrast, gut microbiota dysbiosis promotes chronic hepatic inflammation and contributes to the formation of a pro-tumor microenvironment (12). Underlying liver diseases, such as alcoholic liver disease, can impair intestinal barrier integrity, allowing bacterial products to enter the liver via the gut-liver axis and activate inflammatory signaling pathways. This process induces persistent inflammation and immune dysregulation, promotes immunosuppression in the TME, and ultimately facilitates the malignant transformation of hepatocytes (13, 14). Immune dysregulation in HCC is characterized by excessive inflammation, an immunosuppressive microenvironment, and immune cell dysfunction. Mechanistically, pathogenic microbial products can activate Toll-like receptors (TLRs), leading to excessive activation of innate immune cells and thereby promoting chronic inflammation, liver fibrosis, and carcinogenesis (15). In addition, microbial metabolites can drive hepatic metabolic reprogramming, alter immune cell function, and aggravate immunosuppression (16). The gut microbiota may also interact with intratumoral microbes and immune cells to further remodel the microenvironment and accelerate HCC progression (14).

Against this background, polysaccharides have attracted increasing attention as natural prebiotics because they can selectively promote the growth and metabolic activity of beneficial intestinal bacteria. Owing to their resistance to gastric acid degradation and their selective fermentation in the intestine, polysaccharides enable targeted regulation of gut microbiota composition and function. They can also exert immunomodulatory effects through interactions with intestinal immune cells via pattern recognition receptors (PRRs), including TLRs (17). The structural characteristics of polysaccharides, such as molecular weight and glycosidic bond type, are associated with the abundance of specific bacterial taxa, including Bacteroides and Parabacteroides (18). For example, ginseng polysaccharides promote the growth of Bacteroides and Parabacteroides, whereas dendrobium polysaccharides selectively enhance the growth of Bacteroides, thereby contributing to intestinal microbiota optimization (19). Moreover, their biocompatibility and low immunogenicity make polysaccharides promising candidates for microbiota-based intervention (20). This review focuses on the interaction between polysaccharides and the gut microbiota in the regulation of the HCC immune microenvironment. We first summarize how the gut microbiota shapes the HCC TME through the gut-liver axis and four major metabolic pathways involving bile acids, SCFAs, LPS, and tryptophan. We then discuss how polysaccharides regulate gut microbiota in a structure-dependent manner and how polysaccharide-gut microbiota crosstalk may reshape the immunosuppressive microenvironment in HCC through key pathways, including TLR/NF-κB, bile acid-FXR, and PD-1/PD-L1. Finally, we highlight the potential value of nanopolysaccharides, the etiology-specific heterogeneity of polysaccharide efficacy in HCC, and the major challenges and translational prospects of polysaccharide-based immunotherapy.

## 2 Direct regulation of the hepatic cancer immune microenvironment by the gut microbiota

The gut microbiota directly influences the HCC immune microenvironment through two main mechanisms. One is the activation of innate immune receptors by microbial metabolites. The other is the subsequent modulation of innate immune cell function.

### 2.1 Activation of innate immune receptors by gut microbiota metabolites

**Figure 1:**
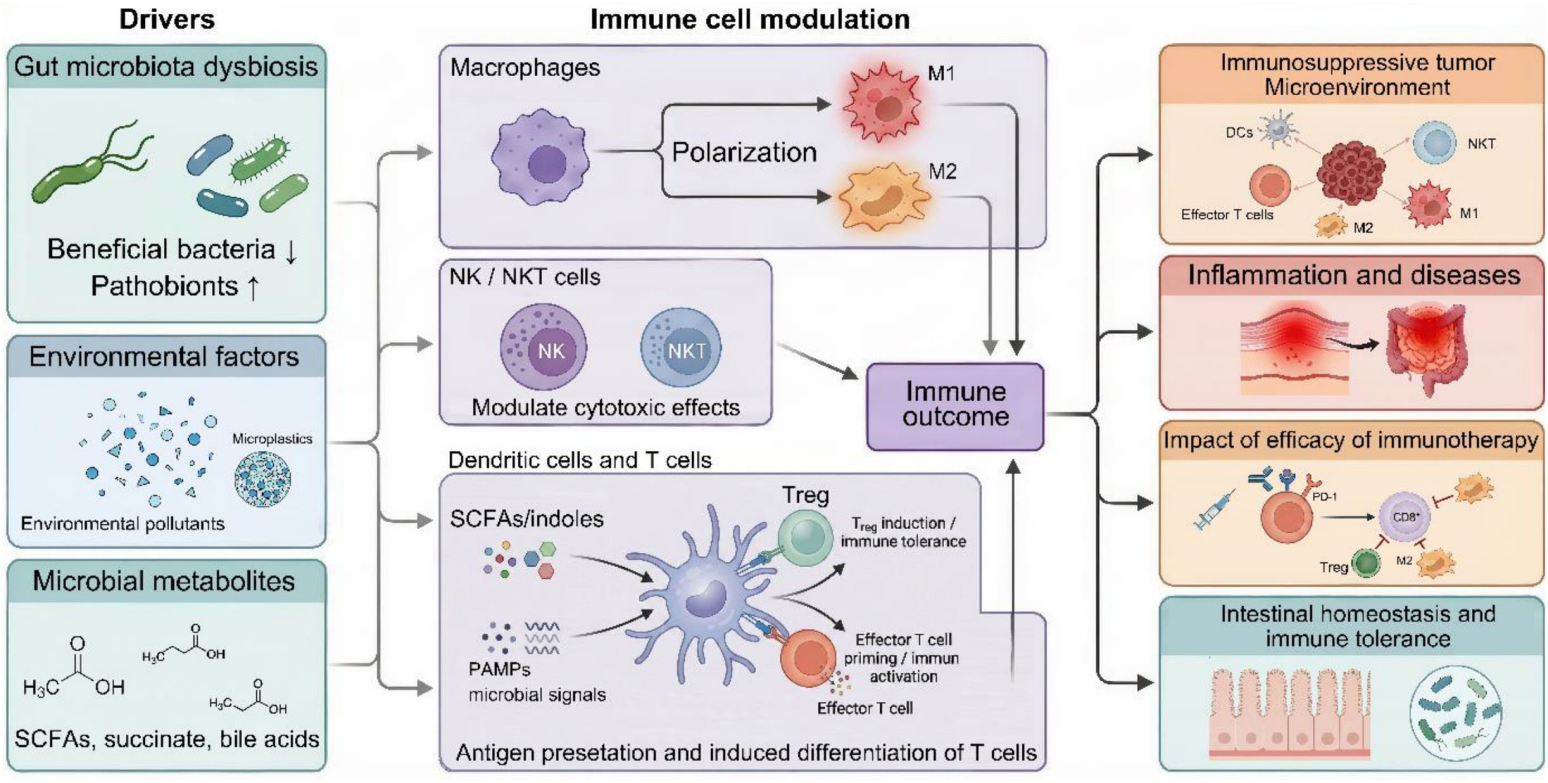
The regulatory mechanisms of gut microbiota on immune modulation. Gut microbiota-related dysbiosis, environmental factors (e.g., microplastics), and microbial metabolites (e.g., succinate) can act on various immune cells: they regulate M1/M2 polarization of macrophages, the cytotoxic/antitumor effects of NK/NKT cells, as well as antigen presentation by DCs and the induced differentiation of regulatory T cells. These processes interact to modulate immune outcomes, thereby impacting the efficacy of immunotherapy, inducing inflammation and diseases, forming an immunosuppressive tumor microenvironment, and maintaining intestinal homeostasis and immune balance.

Gut microbiota-derived metabolites can interact with pattern recognition receptors expressed by hepatocytes and immune cells, including Toll-like receptors (TLRs). These metabolites activate the TLR/NF-κB signaling pathway and induce the release of pro-inflammatory cytokines (**Figure 1**), thereby contributing to the establishment of an immunosuppressive tumor microenvironment in HCC (21, 22). This metabolite-PRR-signaling axis represents an important pathway through which polysaccharide-mediated modulation of the gut microbiota may influence HCC, as polysaccharides can alter the production of microbial metabolites by reshaping gut microbiota composition (23).

### 2.2 Activation and regulatory role of innate immune cells

Activation of innate immune receptors by microbial metabolites ultimately leads to functional changes in innate immune cells, which are central to the regulation of the HCC immune microenvironment. Macrophages are important innate immune cells linking gut microbiota dysbiosis to systemic inflammation and TME remodeling. The gut microbiota can regulate macrophage differentiation through microbial metabolites and molecular signals. For example, microbiota dysbiosis, such as an increased abundance of Escherichia coli in the inflamed colon, can induce activation of inflammatory macrophages in intestinal and extraintestinal tissues and exacerbate inflammation (24). In addition, the gut microbiota can influence macrophage polarization and contribute to the replenishment of the intestinal resident macrophage pool, thereby helping maintain intestinal homeostasis (25). Intestinal epithelial cells, which form the interface between the microbiota and macrophages, also participate in this regulatory process through signal transduction and microbial recognition (26). Moreover, microbial metabolites such as succinate can activate intestinal mucosal macrophages and dendritic cells (DCs), thereby contributing to immune homeostasis through regulation of microbiota-host interactions (27).

Natural killer (NK) cells and natural killer T (NKT) cells play important roles in antitumor immunity, and their functions can be modulated by the gut microbiota through multiple pathways (28). The microbial community can affect the response to anti-PD-1 immunotherapy in advanced melanoma by regulating NK cell activity, and microbiota remodeling may enhance NK cell-mediated tumor cell killing (28). The differentiation and function of NKT cells, which share characteristics of both innate immune cells and T cells, are also regulated by microbial metabolites. Accordingly, gut microbiota dysbiosis may alter NKT-cell activity and thereby contribute to disease development (29, 30).

As antigen-presenting cells, dendritic cells (DCs) bridge innate and adaptive immunity. The gut microbiota can activate intestinal DCs through microbial metabolites, promote their maturation and antigen-presenting capacity, and thereby regulate local immune responses (31). In colorectal cancer (CRC), the gut microbiota can also contribute to the formation of an immunosuppressive TME by altering DC recruitment, activation, and function (32). In addition, the microbiota may induce the differentiation of tolerogenic DCs and regulate regulatory T cells (Treg) through pathways such as interleukin (IL)-2/IL-2R, thereby maintaining intestinal immune tolerance (33).

## 3 Pro-carcinogenic effects of microbiota dysbiosis in HCC

**Figure 2:**
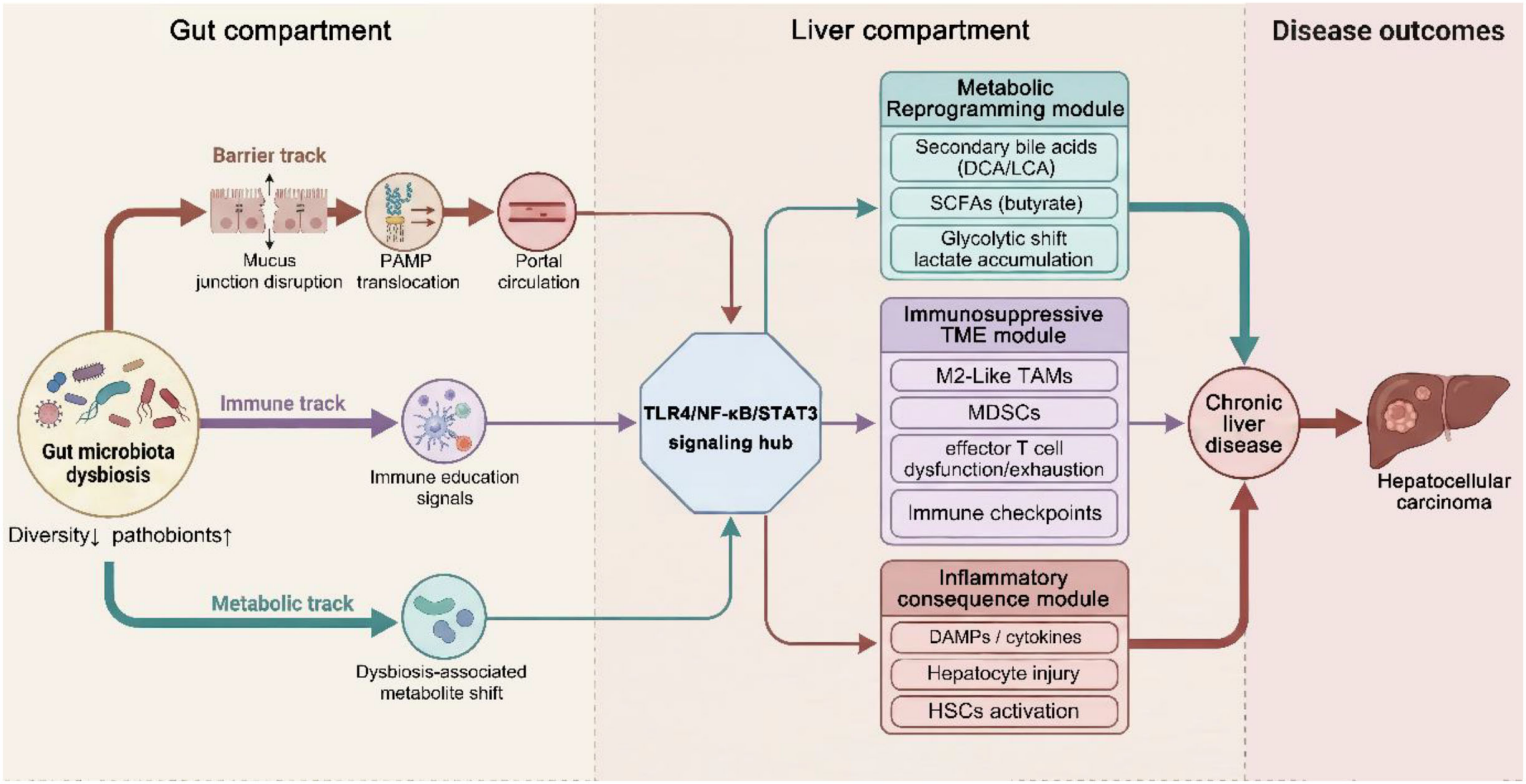
The immunometabolic regulatory mechanisms of gut microbiota dysbiosis in the development of HCC. Gut microbiota dysbiosis can trigger metabolic reprogramming, affecting bile acid metabolism, SCFAs production, and glycolysis, thereby activating signaling pathways such as NF-κB and STAT3. It also induces immune dysregulation, including the activation of M2 macrophages, functional inhibition of effector T cells, upregulation of inflammatory cytokines (e.g., TNF-α, IL-6), and regulation of TLR4 signaling and immune checkpoints. In addition, dysbiosis disrupts the intestinal barrier, allowing LPS to enter the liver via portal circulation and promote the development of chronic hepatitis and liver fibrosis. It also modulates MDSCs to shape an immunosuppressive microenvironment, ultimately participating in the progression of HCC.

As an important component of the human microecosystem, gut microbiota homeostasis is closely associated with the progression of various liver diseases and plays an important role in the initiation and development of HCC (**Figure 2**). Recent studies suggest that microbiota dysbiosis does not arise from a single factor. Instead, it contributes to the pathological progression of HCC through multiple interconnected pathways. Among these, the gut-liver axis serves as a key route for material exchange and signal transmission between the intestine and the liver, and it plays an important role in mediating the effects of dysbiosis on HCC development.

### 3.1 Immune response regulation mechanism

Microbiota dysbiosis modulates hepatic immune responses via the gut-liver axis to drive HCC initiation and progression: it induces hepatic M2 macrophage polarization and effector T cell suppression in Nlrp6-deficient mice, accelerating the malignant transformation of inflammatory/fibrotic liver disease (34, 35); it activates pro-inflammatory cytokines such as interleukin-6 and tumor necrosis factor-α, and induces the generation of inflammatory macrophages mediated by pathogenic bacteria such as Escherichia coli (24, 36, 37); and impairs gut-liver immune homeostasis, leading to reduced efficacy of PD-1/PD-L1 inhibitors (16, 38).

### 3.2 Intestinal barrier destruction mechanism

Microbiota dysbiosis impairs gut-vascular barrier (GVB) integrity and increases intestinal permeability, enabling bacterial translocation and entry of metabolites (e.g., LPS) into the hepatic portal circulation: LPS activates Kupffer cells and hepatic stellate cells via TLR4 to induce liver fibrosis and hepatocarcinogenesis (39); accumulated bacterial genotoxins cause hepatocyte mitochondrial dysfunction, DNA damage and epigenetic-driven abnormal proliferation; meanwhile, microbiota translocation modulates hepatic MDSCs to form an immunosuppressive TME, facilitating HCC immune evasion (40, 41).

### 3.3 Tumor microenvironment and metabolic reprogramming mechanism

GVB damage and microbiota translocation reshape the HCC TME and drive metabolic reprogramming: dysbiotic microbiota alters hepatic immune cell composition, releases pro-inflammatory molecules (e.g., IL-1β) to form a persistent inflammatory TME, and promotes angiogenesis and invasion (40); it also disrupts bile acid/SCFA metabolism, activates oncogenic pathways (NF-κB, STAT3) and shifts hepatic energy metabolism to glycolysis, supporting HCC cell proliferation (42). In addition, the degree of microbiota disorder may be related to HCC tumor stage. The abnormal enrichment of specific microorganisms (e.g., Escherichia coli) in patients can further exacerbate liver damage and carcinogenesis via molecular mimicry or cross-reaction mechanisms, but the specific connection still needs further research and verification (43–45).

In summary, microbiota dysbiosis promotes HCC progression via three core interrelated mechanisms, all of which are key targets for polysaccharide intervention: (1) activating the TLR4/NF-κB pathway via the gut-liver axis to induce hepatic immune cell dysfunction and reduce ICI efficacy; (2) destroying the intestinal barrier to trigger bacterial translocation and LPS-mediated chronic liver inflammation; (3) reshaping the immunosuppressive TME and driving metabolic reprogramming (bile acid/SCFA metabolism disorder). Polysaccharides can target these mechanisms by regulating gut microbiota composition and metabolism, thus becoming a potential strategy for HCC prevention and treatment (**Table 1**).

**Table 1:** Gut microbiota metabolic axes and their impacts on the immune microenvironment of HCC.

Microbiota node	Metabolite/signal	Immune component	Immune microenvironment	References
BAs metabolic axis				
Gut microbiota (esp. bile salt-tolerant, e.g., some Bacteroides)	Secondary BAs (e.g., DCA, LCA) ↑	CXCR6^+^ NKT cells ↓	Secondary BAs downregulate CXCL16 in liver sinusoidal endothelial cells via FXR/TGR5, which impairs NKT cell recruitment, weakens immune surveillance, and promotes HCC.	(46)
Gut microbiota	Secondary BA (LCA) ↑	CD8^+^ T cells (dysfunctional)	Accumulated LCA in the TME induces the ER-stress-CHOP axis in CD8^+^ T cells, leading to exhaustion and enhanced immune suppression.	(47)
Intervened Bacteroides/Lactic acid bacteria ↑	Primary BAs ↑, Secondary BAs ↓ (via BSH activity ↑)	NKT cells (IFN-γ^+^) ↑	Inhibits primary-to-secondary BAs conversion, activates the IFN-γ axis of liver NKT cells, and exerts an anti-tumor immune effect.	(48)
Akkermansia muciniphila	Bile acids ↓	CXCR6^+^ NKT cells ↑	Supplementation of A. muciniphila increases NKT cell number, blocking NASH progression to HCC.	(49)
Bifidobacterium breve (harboring bsh gene)	Deconjugated taurocholic acid (TCA) ↓	CD8^+^ T cells (infiltration restored)	BSH-expressing strains decouple TCA, relieving ERK signaling inhibition and restoring CD8^+^ T cell infiltration in tumors.	(50)
SCFAs metabolic axis				
Butyric acid-producing bacteria	Butyrate (butyrate salt) ↑	CD8^+^ T cells (CTL, cytotoxic T lymphocyte) ↑, TAM (immunosuppressive) ↓	Butyrate, as an histone deacetylases (HDAC, histone deacetylase) inhibitor, promotes MYC degradation or activates NF-κB via TLR5/GPR109A, enhancing CD8^+^ CTL cytotoxicity.	(51) (52),
Gut microbiota	Butyrate (butyrate salt)	NK cells ↑	Butyrate upregulates the tumor chemokine CXCL11 via the HDAC inhibition-STAT pathway, enhancing NK cell migration and infiltration.	(53)
Lactobacillus reuteri/Gut microbiota	Acetate (acetate salt) ↑	ILC3 ↓, IL-17A ↓	Acetate increases Sox13 acetylation via HDAC inhibition, inhibiting IL-17A production by ILC3 and reducing pro-tumor inflammation.	(54)
Gut microbiota	Propionate (propionate) ↑	CD8^+^ T cells (infiltration) ↑, PD-L1 (on tumor cells) ↓	Propionate activates AMPK via GPR43/FFA2, reprograms tumor metabolism, promotes CD8^+^ T cell infiltration, and downregulates PD-L1.	(55)
Bifidobacterium genus	Isobutyrate (isobutyrate salt) ↑	CD8^+^ T cells (IFN-γ^+^) ↑	Isobutyrate inhibits the JAK/STAT3 pathway, enhances CD8^+^ T cell function, and synergizes with αPD-1 therapy.	(56)
Gut microbiota	Butyrate (butyrate salt)	TAM (M2c type) ↑	Butyrate drives TAM polarization to M2c via the GPR109A-PKA-PPARγ-MertK axis, secreting IL-10/TGF-β and promoting immunosuppression.	(57)
LPS and other bacterial components				
Gram-negative bacteria	LPS	TAM (M2 type) ↑	LPS activates TLR4 and downstream NF-κB/STAT3 signaling in TAMs, inducing their polarization to M2 type and promoting HCC cell invasion and migration.	(58) (59),
Gram-negative bacteria (in HBV infection context)	LPS	Kupffer cells (innate immune function) ↓	HBV HBeAg inhibits LPS-induced NLRP3 inflammasome activation via the TLR4-NF-κB pathway in Kupffer cells, weakening their innate immune scavenger function.	(60)
Gram-positive bacteria (e.g., Clostridial cluster XIVa)	Lipoteichoic acid (LTA)	Regulatory T cells ↑	LTA accumulates in hepatic stellate cells (HSCs) via the gut-liver axis, releases IL-33 through the TLR2-GSDMD signaling pathway, and thus activates and recruits Treg to form an immunosuppressive microenvironment.	(61)
Tryptophan metabolic axis				
Phocaeicola vulgatus (enriched in non-responders to immunotherapy)	Indole acetic acid (IAA) ↓	CD8^+^ T cells (IFN-γ^+^, GzmB^+^) ↓	This bacterium depletes the tryptophan metabolite IAA, impairing CD8^+^ T cell function and inducing resistance to PD-1 inhibitors.	(62)
Tumor and immune cells	Kynurenine ↑ (via IDO1 enzyme)	MDSCs ↑	Activation of the IDO1-kynurenine-AhR axis promotes MDSC infiltration in the TME, forming strong immune suppression.	(63)
Intervention (IDO inhibitor)	Kynurenine ↓	CD8^+^ T cells ↑, Treg ↓, IFN-γ ↑, TNF-α ↑	Pharmacological inhibition of IDO1 reverses kynurenine-mediated immune suppression, restores T cell function, and synergizes with chemotherapy.	(64)
Akkermansia muciniphila	LPS ↓, Bile acids ↓	MDSC ↓, M2 TAM ↓, T cell infiltration ↑	Supplementation of A. muciniphila alleviates immune suppression in MAFLD-related HCC, significantly improving PD-1 inhibitor efficacy.	(65)
Bifidobacterium longum	(Unknown metabolite)	Bacteria-specific CD8^+^ T cells ↑, Treg ↓	In HBV-related HCC patients, a high frequency of B. longum-reactive CD8^+^ T cells correlates with better disease-free survival.	(66)
Antibiotics (vancomycin/colistin)	Microbiota dysbiosis	CD4^+^ T cells ↓, IFN-γ ↓, IL-2 ↓	Broad-spectrum antibiotics disrupt gut microbiota homeostasis, reducing key helper T cells and cytokines, thus impairing PD-1 inhibitor efficacy.	(67)
Fecal microbiota transplantation (FMT, from responders)	Microbiota reshaping	IFN-γ ↑, TNF-α ↑, IL-6 ↓	FMT from immunotherapy responders remodels the gut microbiota, reactivates anti-tumor inflammatory pathways, and reverses PD-1 resistance.	(68)

↑ Increase, ↓Decrease

Polysaccharides regulate immune responses via multi-target, multi-pathway mechanisms to enhance anti-tumor immunity, and are thus incorporated into a new generation of cancer immunotherapy strategies to boost anti-tumor effects by regulating immune cell activity (e.g., DCs) or promoting T cell infiltration (69). For example, in HCC models, the immunomodulatory effect of polysaccharides has been verified: they can promote DC activation and cytotoxic T cell infiltration, thereby exerting anti-tumor effects in the microenvironment (70). In addition, the therapeutic potential of polysaccharides has been confirmed in preclinical models—for instance, in human HCC and breast cancer organoid models, polysaccharides exhibit therapeutic and immunomodulatory effects, and can enhance anti-tumor immunity via a cascade amplification mechanism (71), indicating that polysaccharides have broad application prospects in immunoregulation.

## 4 Regulation of HCC immunity by polysaccharide-gut microbiota interaction

**Figure 3:**
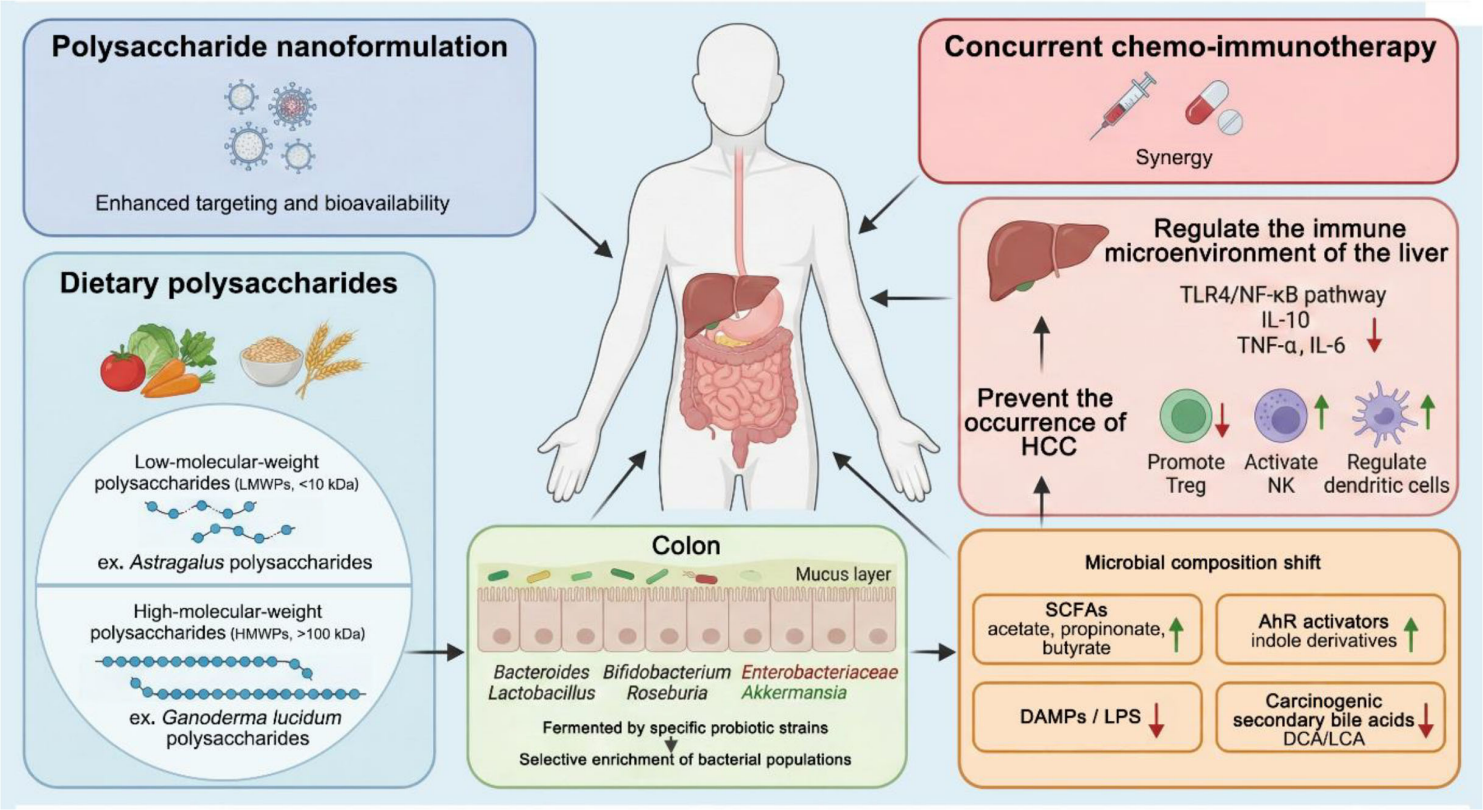
The immunometabolic regulatory mechanism of dietary fiber-derived polysaccharides in preventing HCC via the gut-liver axis. Dietary fiber supplies polysaccharides of varying molecular weights (e.g., astragalus and Ganoderma lucidum polysaccharides), which are fermented by specific gut probiotic strains to enrich beneficial bacterial populations. It also modulates gut metabolism: boosting beneficial metabolites (e.g., SCFAs) and reducing harmful substances (e.g., LPS, carcinogenic bile acids). These alterations regulate the liver’s immune microenvironment: inhibiting the TLR4/NF-κB pathway, modulating immune cells (promoting Treg, activating NK cells), and balancing inflammatory cytokines (increasing IL-10, decreasing TNF-α/IL-6). Ultimately, these regulations prevent HCC, and strategies like polysaccharide nanonization, bioavailability enhancement, and combination with chemoimmunotherapy can reinforce these effects.

Polysaccharides interact with the gut microbiota to regulate HCC immunity via a cascade of events: from selective modulation of microbiota composition, to microbial metabolism of polysaccharides into immunologically active metabolites, and finally to the reshaping of HCC immune microenvironment via metabolite-mediated pathway regulation and metabolite balance adjustment; nanopolysaccharides further optimize this regulatory process by enhancing targeting efficiency (**Figure 3**).

### 4.1 Specific regulation of intestinal microbiota composition by polysaccharides

Polysaccharides specifically regulate the composition of the gut microbiota through a structure-glycosidase-microbiota causal axis. The chemical structures of polysaccharides determine their recognition and utilization by specific gut microbiota. This matching depends on the compatibility between polysaccharide structures and the glycoside hydrolase (GH) families of bacteria. The chemical characteristics of polysaccharides include glycosidic bond type, degree of branching and molecular weight. High-molecular-weight polysaccharides with α-1, 4 glycosidic bonds (molecular weight >100 kDa) are recognized by the GH13 family of Bifidobacterium and Lactobacillus, which promotes the proliferation of these bacteria (72); β-1, 3/1, 6-glycosidic bond low-molecular-weight polysaccharides (<10 kDa) are hydrolyzed by the GH5 family of Prevotella/Bacteroides for selective enrichment (18, 73).

Meanwhile, polysaccharides can achieve selective enrichment of specific microbiota—for example, astragalus polysaccharides (APS) with a unique β-1, 4-glucan backbone can be specifically degraded by the GH43 family of Akkermansia muciniphila, thus promoting the enrichment of this bacterium, which in turn degrades intestinal mucus layer polysaccharides, produces SCFAs and enhances intestinal barrier function (74); ganoderma lucidum polysaccharides containing β-1, 3/1, 6-glucan are recognized by the GH16 family of Roseburia, driving the proliferation of this butyrate-producing bacterium (75).

### 4.2 Production of immunologically active substances by gut microbiota metabolizing polysaccharides

Polysaccharide-mediated beneficial bacterial enrichment drives microbial metabolic remodeling to produce immunologically active substances, with SCFAs as the core effector: polysaccharides are fermented via the gut microbiota’s carbohydrate metabolism pathways (Embden-Meyerhof-Parnas, pentose phosphate) to generate acetate, propionate and butyrate, whose type and yield depend on polysaccharide structure and bacterial metabolic enzymes (24, 76). Additionally, Lactobacillus-derived tryptophan metabolites (e.g., indole-3-carboxaldehyde) activate AhR to reduce intrahepatic inflammation and enhance CD8^+^T cell anti-tumor cytotoxicity (45).

### 4.3 Impact of polysaccharide-microbiota interaction on the HCC immune microenvironment

These immunologically active substances generated by microbial metabolism of polysaccharides ultimately act on the HCC immune microenvironment via the gut-liver axis, and the regulatory effects of polysaccharide-microbiota interaction are mainly reflected in two key aspects: first, polysaccharides reduce the proportion of LPS-producing bacteria (e.g., Enterobacteriaceae) by increasing probiotic abundance, thereby reducing LPS entry into the blood—LPS binds to hepatic TLR4 to activate the NF-κB pathway, promoting the release of IL-6 and TNF-α to drive HCC progression, while SCFAs can directly inhibit TLR4 signaling and reduce intrahepatic inflammation (77); second, polysaccharides can enhance immune checkpoint therapy efficacy—for example, wolfberry polysaccharides enrich Bifidobacterium, promote intestinal DC maturation, increase IL-12 secretion, and thus improve the therapeutic effect of PD-1/PD-L1 inhibitors on HCC (78).

### 4.4 Regulation of the balance of microbiota-related metabolites

In addition to directly regulating immune cells and inflammatory pathways, polysaccharide-microbiota interaction exerts anti-hepatoma effects by adjusting the balance of microbiota-related metabolites. On one hand, polysaccharides can reduce the production of microbiota-derived trimethylamine (TMA), inhibiting the conversion of TMA to trimethylamine N-oxide (TMAO) by the hepatic FMO3 enzyme—TMAO promotes HCC metastasis by activating the NLRP3 inflammasome, and polysaccharide intervention can reverse this effect; on the other hand, polysaccharides activate FXR receptors by increasing the proportion of SCFA-producing bacteria, regulate the expression of bile acid synthetase, and reduce the accumulation of carcinogenic bile acids (e.g., deoxycholic acid, DCA), thus decreasing DNA damage and hepatic stellate cell activation (79, 80).

The gut microbiota ferments polysaccharides to produce bioactive metabolites such as SCFAs (e.g., acetate, propionate, butyrate) and bile acid derivatives (e.g., deoxycholic acid, lithocholic acid). These metabolites enter the liver microenvironment via the “gut-liver axis” and directly affect the HCC immune response—among them, SCFAs enter the liver via the portal circulation to regulate intrahepatic immune cell function (81–83), and bile acid metabolites (e.g., deoxycholic acid) are formed by microbiota modifying primary bile acids and can promote inflammatory responses in the HCC microenvironment. SCFAs have a bidirectional regulatory effect on HCC immune cells: they can activate the Foxp3 gene by inhibiting HDAC to promote Treg differentiation, reduce pro-inflammatory factor release, and inhibit excessive local inflammatory responses in HCC (84); they can also activate NK cells via GPR43 receptors to enhance their killing ability against HCC cells (85); they can reduce excessive T cell activation by downregulating DC pro-inflammatory factors (e.g., IL-12) to maintain immune homeostasis (86); at the same time, they reduce pro-inflammatory factor secretion by M1-type macrophages by inhibiting the NF-κB pathway and block their supportive effect on HCC growth (87).

In addition, some microbial metabolites can alter anti-tumor immune responses by affecting immune checkpoint molecules. For example, SCFAs can upregulate immunosuppressive molecules like CTLA-4 and PD-1 to promote immune tolerance (this mechanism may also inhibit excessive immune damage in HCC) (21), while bile acid metabolites (e.g., deoxycholic acid) promote PD-L1 expression by activating hepatic stellate cells, helping HCC cells evade immune surveillance. Polysaccharide metabolites can also reshape the HCC immune microenvironment in multiple ways: their produced SCFAs can promote the expression of intestinal epithelial tight junction proteins, reduce intestinal endotoxin (e.g., LPS) entry into the blood, and reduce hepatic TLR4 activation to weaken pro-inflammatory pathways (16); microbial metabolites can activate the host antioxidant enzyme system (e.g., SOD) to reduce reactive oxygen species damage to immune cells (88, 89); simultaneously, polysaccharide-mediated SCFAs increase the secretion of the anti-inflammatory factor IL-10 and inhibit pro-carcinogenic factors IL-6 and IL-1β, thus improving the immune microenvironment (90).

### 4.5 Immunoregulatory role of nanopolysaccharides in HCC

Building on the core regulatory mechanisms of polysaccharide-microbiota interaction in HCC immunity, nanotechnology has been applied to optimize the delivery and efficacy of polysaccharides, leading to the emergence of nanopolysaccharides as a promising research direction. Nanopolysaccharides are engineered by modifying natural polysaccharides (alginate, plant extracts) into nanoparticles/micelles, which significantly improve tumor targeting and bioavailability. Their mechanisms for regulating the immune microenvironment in HCC treatment focus on three aspects: first, targeted delivery and immune enhancement. Nanopolysaccharide systems can specifically target tumor sites and overcome immune microenvironment barriers. For example, “biotinylated aldehyde alginate-doxorubicin nanomicelles” (BEA–Cn–DOX–M) combine the natural immune activation effect of alginate with the chemotherapeutic effect of doxorubicin, can enhance the functions of immune cells like macrophages and T cells, induce cancer cell apoptosis, and reduce the activity of tumor-associated fibroblasts and immunosuppressive cells to reshape the TME (91); polysaccharide-based nanoplatforms can also be used for “cancer chemotherapy-innate immunotherapy”, which can promote the recruitment and activation of NK cells and exert a synergistic regulatory effect on the HCC immune microenvironment (82).

Second, regulation of the gut-liver axis. Nanopolysaccharides can interact with the gut microbiota, and nanodrug delivery systems (NDDS) can affect microbial metabolites (e.g., SCFAs) via this axis to regulate intrahepatic immune responses. Nanopolysaccharides can improve intestinal barrier function and increase the production of beneficial microbial metabolites (e.g., SCFAs) to inhibit HCC progression (82, 92), and this “active targeting” mechanism also helps balance the immune microenvironment.

However, most current related studies are in the preclinical stage. Nanopolysaccharides have advantages such as high biocompatibility, low toxicity, and the ability to combine with therapies like immune checkpoint inhibitors (91), but the uniqueness of the HCC immune microenvironment (e.g., immune tolerance) increases treatment difficulty. Although nanoparticles can partially overcome this obstacle, their targeting efficiency still needs optimization; overall, nanopolysaccharides as a new delivery system have clear potential in regulating the HCC immune microenvironment, but more clinical data are needed for support.

Polysaccharides in natural products are an important direction in the research of anti-inflammatory pathways. Their regulatory signaling pathways and their role in the immune microenvironment of HCC have become core research topics in the field of natural products against HCC. Relevant studies focus on the molecular mechanisms of polysaccharides targeting key anti-inflammatory/immune-related signaling pathways such as NF-κB, MAPK, and JAK/STAT. Meanwhile, they analyze the specific effects of polysaccharides on immune cells (T cells, macrophages, DCs, etc.), cytokine expression, and remodeling of the immunosuppressive microenvironment in the HCC immune microenvironment, laying a theoretical foundation for the research and development of polysaccharide-based anti-HCC immunomodulators (**Table 2**).

**Table 2:** Polysaccharides: signal pathways, impacts on HCC immune microenvironment.

Polysaccharide	Pathways/cytokine changes	Immune microenvironment	References
Cinobufagin Polysaccharide/Hua’er Polysaccharide (HP)	Alters intestinal flora structure, upregulates CDCA; inhibits M2 polarization, promotes M1 polarization; ↑TNF-α, ↑IFN-γ, ↑IL-6, ↑IL-12p40	Remodels the immunosuppressive microenvironment via the gut-liver axis and enhances anti-tumor immune response.	(93)
Phyllanthus emblica Pectin Polysaccharide (PEP-1)	Activates NF-κB and MAPK signaling pathways; induces M2→M1 polarization; ↑p-NF-κB, ↑p-MAPK; ↑IL-12, ↑TNF-α	Converts “cold” tumors to “hot” tumors, enhances M1 macrophage infiltration, and induces tumor cell apoptosis.	(94)
Radix Tinosporae Polysaccharide (RTP-W)	Activates the Akt signaling pathway; downregulates PD-1 expression; ↑IL-2, ↑IFN-γ	Relieves CD8^+^T cell exhaustion, restores their proliferation and cytokine secretion capabilities, and enhances tumor killing.	(95)
APS	1. Promotes STAT5 phosphorylation and increases the proportion of CD122^+^CXCR3^+^PD-1^-^ memory T cells. 2. Upregulates miR-133a-3p, targets the MSN gene, and promotes tumor cell PD-L1 degradation.	1. Synergizes with CAR-T therapy to enhance its persistence and cytotoxicity in solid tumors. 2. Reduces the immune escape ability of tumor cells.	(96, 97),
Ganoderma Lucidum Polysaccharide (GLPS)	1. Activates MAPK (MEK/ERK) and NF-κB signaling pathways to promote M1 polarization (↑CD86, ↑iNOS, ↑IL-12). 2. Inhibits the TGF-β/Smad2/3 signaling pathway and downregulates Foxp3 expression.	Promotes M1 macrophage infiltration while reducing immunosuppressive Treg cells, synergistically enhancing anti-tumor immunity.	(98)
Grifola frondosa Polysaccharide-Protein Complex (GFG-4)	Activates the TLR4-NF-κB signaling pathway; regulates intestinal flora and increases butyrate production; ↑NK cell activity, ↑IFN-γ, ↑IL-2	Enhances systemic and local anti-tumor immunity through the dual pathways of “direct TLR4 activation + intestinal flora metabolites”.	(99, 100),
Fucoidan	Inhibits NF-κB (p65) phosphorylation, downregulates CCL22 secreted by M2 macrophages; activates NK cells; inhibits the PI3K/AKT/mTOR pathway.	Reduces the recruitment of Treg cells to the tumor area; activates NK cell killing; inhibits angiogenesis and inflammation.	(101, 102),
Chitosan (and its derivatives)	Bidirectionally regulates macrophage phenotype (low concentration promotes M1, high concentration promotes M2); downregulates Tim-3 expression as a carrier.	Can directly regulate macrophages and serve as a nanocarrier for siRNA or drug delivery, enhancing CTL infiltration and inhibiting immune checkpoints.	(103, 104),
Alginate (and its hydrogels)	Neutralizes the acidic tumor microenvironment; activates the STING pathway as a carrier; induces immunogenic cell death (ICD, immunogenic cell death).	Local application can remodel the physical and chemical microenvironment, activate innate immune signals, and induce a strong systemic anti-tumor immunity (abscopal effect).	(105, 106),
Bacterial Extracellular Polysaccharide (EPS)	Promotes the expansion of IFN-γ^+^CD8^+^T cells; synergizes with sorafenib to induce ferroptosis (iron efflux) in tumor cells.	Enhances T cell function and synergizes with targeted drugs to induce new tumor cell death modes, overcoming drug resistance.	(107)
Echinacea Purpurea Polysaccharide (EPP)	Improves the intestinal barrier and reduces LPS leakage; inhibits the hepatic TLR4-NF-κB signaling pathway; ↓IL-6, ↓MMP-2	Inhibits the growth and invasion of HCC by repairing the intestinal barrier and reducing systemic inflammation.	(108)
Selenylated Modified Polysaccharides (e.g., FCP-SeNPs, PAP-SeNPs)	Activates the TLR4/MyD88/NF-κB signaling pathway; ↑NO, ↑TNF-α, ↑IL-12; ↑CD4^+^/CD8^+^T cell infiltration	Compared with original polysaccharides, selenylation modification significantly enhances immune activation ability and anti-tumor effect.	(109)

## 5 Key signaling pathways for the regulation of HCC immunity by polysaccharide-gut microbiota interaction

Polysaccharide-gut microbiota interaction regulates HCC immunity by targeting multiple core signaling pathways, which are interconnected and synergistically reshape the HCC immune microenvironment—these pathways include the classic inflammatory pathway TLR/NF-κB, the metabolic regulatory axis bile acid-FXR, the microbiota metabolite-driven SCFAs axis, and the immune checkpoint pathway PD-1/PD-L1 that is critical for immunotherapy efficacy.

### 5.1 TLR/NF-κB pathway

The TLR/NF-κB pathway is a classic inflammatory signaling axis. It mediates hepatic inflammation induced by the gut microbiota and the formation of the HCC tumor microenvironment. Polysaccharides regulate this pathway through two ways: microbiota-dependent indirect inhibition and structure-mediated direct suppression. This regulatory effect has a critical dose-dependent characteristic. The gut microbiota and its metabolites act on pattern recognition receptors such as Toll-like receptors. They activate the downstream NF-κB signaling pathway and trigger the innate immune response of the liver. Toll-like receptors are key molecules in hepatic immune regulation. They are involved in inflammatory responses, epithelial regeneration and carcinogenesis (15). In HCC, the gut microbiota can also regulate the TME via the TLR/NF-κB pathway, affecting immune cell infiltration and function (110).

Polysaccharides like mushroom polysaccharides and seaweed polysaccharides can indirectly inhibit TLR/NF-κB pathway activation by changing gut microbiota composition and metabolite (e.g., SCFAs) production. Among these, SCFAs can reduce liver inflammation by downregulating TLR expression and inhibiting NF-κB phosphorylation (111, 112); microbiota changes regulated by polysaccharides can further inhibit the release of TLR/NF-κB-mediated pro-inflammatory factors (e.g., IL-1β, IL-6) and promote the expression of anti-inflammatory factors (e.g., TGF-β), improving the HCC immunosuppressive microenvironment (100). In addition, some polysaccharides (e.g., herbal polysaccharides) can directly inhibit the expression and phosphorylation of key proteins (e.g., TLR4, p65) in the TLR4/NF-κB pathway, blocking NF-κB nuclear translocation to reduce liver inflammation and damage (113, 114); this direct effect is related to polysaccharide structural characteristics such as glycosidic bond type (74).

In HCC immunotherapy, the gut microbiota regulates HCC sensitivity to immune checkpoint inhibitors (ICB) via the TLR/NF-κB pathway: pathway activation may lead to immune escape and ICB resistance, while pathway inhibition can enhance anti-tumor immune responses (13). Targeting the TLR/NF-κB pathway (e.g., using the TLR4 inhibitor TAK-242) combined with polysaccharide intervention can synergistically enhance anti-tumor effects. Regulating the microbiota and inhibiting the TLR/NF-κB pathway via polysaccharides can delay the malignant transformation of chronic liver diseases to HCC (16); this polysaccharide intervention strategy based on the microbiota-TLR/NF-κB axis is expected to improve the response rate of HCC immunotherapy and show good clinical application potential (77).

It is worth noting that the regulation of TLR/NF-κB pathway by polysaccharides has a clear dose dependence, and different polysaccharides have specific safe dose windows, which is a key factor to avoid potential liver damage. Studies have shown that some polysaccharides (such as astragalus polysaccharides, Ganoderma lucidum polysaccharides and other fungi and plant-derived polysaccharides) have a dual regulatory effect on the TLR4/NF-κB pathway: at low to medium doses (such as astragalus polysaccharides 100–200 μg/mL, Ganoderma lucidum polysaccharides 50–100 μg/mL), they can indirectly inhibit the activation of intrahepatic TLR4/NF-κB pathway by reducing the production of LPS from intestinal flora and enriching the bacteria that produce SCFA; while at high doses (such as astragalus polysaccharides >500 μg/mL, Ganoderma lucidum polysaccharides >300 μg/mL), they can directly bind to the TLR4/MD2 complex on the surface of hepatocytes and Kupffer cells, activate the MyD88-dependent NF-κB signaling pathway, promote the release of pro-inflammatory factors such as IL-6 and TNF-α, and then aggravate chronic hepatitis or even induce liver tissue damage (115–117). This dual effect is closely related to the structural characteristics of polysaccharides. Polysaccharides with a high degree of branching have a stronger binding affinity for TLR4, so they are more likely to trigger direct activation at high doses.

### 5.2 Bile acid-FXR axis

The bile acid-FXR axis is a key metabolic-immune signaling pathway that links gut microbiota bile acid metabolism to HCC TME regulation; polysaccharides reshape this axis by modulating gut microbiota to correct bile acid profile disorders and activate FXR-mediated anti-tumor signaling. Beyond the TLR/NF-κB pathway, the bile acid- FXR axis is another core signaling pathway through which polysaccharide-gut microbiota interaction regulates HCC immunity, and it exerts its effects via metabolic reprogramming and receptor-mediated signal transduction. As important gut microbiota regulators, polysaccharides can affect bile acid metabolic homeostasis by reshaping microbiota composition and function: mushroom polysaccharides can regulate dominant intestinal microbiota structure (e.g., increasing beneficial bacteria abundance) to maintain microbial balance (1); seaweed polysaccharides exert microbiota-regulating effects by adjusting cecal and fecal microbiota abundance (118); plant polysaccharides like Semiaquilegia adoxoides pectin can be directly metabolized by the gut microbiota, thus altering microbial structure (103). The gut microbiota mediates the conversion of primary bile acids (e.g., conjugated bile acids) to secondary bile acids (e.g., tauroursodeoxycholic acid, TUDCA) by secreting bile salt hydrolase (BSH). Changes in microbiota composition can directly regulate the bile acid profile—for example, increased abundance of Lactobacillus gasseri or Ruminococcaceae can reduce BSH activity and unconjugated bile acid levels (119); Tongxieyaofang polysaccharides can correct bile acid metabolic disorders by improving microbial imbalance and upregulating Lachnospiraceae abundance (120). This microbiota-dependent bile acid metabolic reprogramming provides a molecular basis for the precise regulation of the downstream FXR signaling pathway.

FXR (a nuclear receptor mainly expressed in the liver and intestine) requires specific bile acid binding for activation: secondary bile acids (e.g., deoxycholic acid) and derivatives (e.g., TUDCA) can bind to FXR, inducing it to form a heterodimer with the retinoid X receptor (RXR). This complex binds to FXR response elements (FXRE) to regulate the transcriptional expression of downstream target genes (121, 122). The interaction between bile acids and FXR is the core mechanism for maintaining bile acid homeostasis. FXR activation can reduce abnormal liver bile acid accumulation by inhibiting the expression of hepatic bile acid synthetase (e.g., CYP7A1) (123, 124). In the HCC pathological microenvironment, FXR usually exerts a tumor-suppressive function; its downregulation is closely related to increased HCC risk, which stems from FXR’s multi-faceted regulation of bile acid metabolism, inflammatory responses, and immune responses (125, 126); specifically, FXR activation can inhibit MYC oncogene expression, regulate β-catenin interaction, and control NF-κB pathway activity, thus inhibiting tumor progression (127, 128). However, bile acid profile disorders (e.g., abnormal elevation of secondary bile acids) can inhibit FXR signal transduction, disrupt the normal interaction between FXR and β-catenin, and promote tumor immune escape and HCC occurrence/development (126), indicating that abnormal bile acid metabolism directly regulates the HCC immune microenvironment via the FXR signaling pathway.

FXR is expressed in immune cells like hepatic macrophages and T cells, and its activation state can directly affect immune cell differentiation fate and cytokine secretion profiles, thus reshaping the HCC TME: bile acids regulate HCC immune responses via the FXR signaling pathway. For example, FXR activation can inhibit NF-κB pathway activity, reduce pro-inflammatory factor (e.g., TNF-α) release, and upregulate anti-inflammatory chemokine expression to construct an anti-TME (119, 121); bile acids like norcholic acid participate in regulating immune responses during HCC progression via FXR-mediated signal transduction (129). In addition, FXR can synergize with TGR5, vitamin D receptor (VDR), etc., to regulate the functional activity of key immune cells (e.g., NK cells, DCs), thus inhibiting tumor growth (130). The gut microbiota-bile acid-FXR axis plays an integrating role in HCC immune regulation: polysaccharides can upregulate intestinal FXR expression by regulating microbiota structure (e.g., increasing Lactobacillus abundance), inhibit NF-κB pathway activity, and reverse the tumorigenic process (121); in colon cancer models, microbiota imbalance-induced bile acid profile changes (e.g., TUDCA accumulation) can inhibit FXR signals, and polysaccharide intervention can block this pro-carcinogenic process (127); in HCC, microbiota-mediated FXR pathway regulation can inhibit tumor growth and immune tolerance by reducing bile acid toxicity and restoring normal FXR function (131).

Polysaccharides reshape the bile acid-FXR signaling pathway via microbiota-dependent mechanisms—this is a key molecular mechanism for their regulation of HCC immunity: plant polysaccharides can regulate bile acid metabolism via the “polysaccharide-gut microbiota-FXR signal axis” to exert anti-inflammatory and anti-tumor effects (132, 133); in chemically induced HCC models, polysaccharides optimize the bile acid profile by regulating microbial balance (e.g., increasing Bacteroides abundance), promote FXR activation, thus inhibiting immunosuppressive factor expression and improving the body’s anti-tumor immune surveillance (134). This interaction network highlights the FXR signaling pathway’s key role as a core bridge connecting polysaccharide-induced microbiota changes and HCC immune regulation—by downregulating the activity of inflammatory pathways (e.g., NF-κB) and upregulating immune checkpoint inhibition-related molecule expression, the anti-tumor effect is ultimately achieved (10). In summary, polysaccharides like mushroom polysaccharides and plant polysaccharides regulate gut microbiota composition and metabolic functions, reprogram the bile acid profile (e.g., increasing beneficial secondary bile acid production), optimize FXR signaling pathway activation state, and ultimately reshape the TME by inhibiting HCC-related inflammatory pathways and regulating immune cell functions; based on the above mechanisms, intervention strategies targeting the gut microbiota-bile acid-FXR axis (e.g., FXR agonists combined with polysaccharide intervention) are expected to become potential HCC treatment directions, and combining microbiota modulation can further enhance therapeutic efficacy (76).

### 5.3 SCFAs axis

The SCFAs axis is the primary effector pathway for polysaccharide-microbiota interaction to regulate HCC immunity, exerting anti-tumor effects via GPCR activation and HDAC inhibition to modulate hepatic immune cells and TME. Complementary to the bile acid-FXR axis, the SCFAs axis serves as the core effector pathway for gut microbiota to metabolize polysaccharides, and it regulates HCC immunity through dual mechanisms of G protein-coupled receptor (GPCR) activation and HDAC inhibition. The structural characteristics of polysaccharides (e.g., plant, fungal, and seaweed polysaccharides) are the core basis for their precise modulation of the gut microbiota. These biological macromolecules can selectively regulate gut microbiota composition and abundance via the specificity of molecular weight, glycosidic bond type, monosaccharide composition, and advanced structure—their enrichment effect on SCFA-producing beneficial bacteria (e.g., Lachnospiraceae, Roseburia) is particularly significant (74, 135); polysaccharides with different structural characteristics are specifically recognized and utilized by the above microbiota, then gradually degraded and fermented via the microbiota’s glycoside hydrolase system, ultimately producing SCFAs dominated by acetate, propionate, and butyrate (136, 137). For example, high-molecular-weight polysaccharides are difficult to be hydrolyzed by upper gastrointestinal enzymes and can reach the colon intact to be preferentially utilized by Lachnospiraceae, while fungal polysaccharides containing β-1, 3/1, 6-glycosidic bonds are more easily recognized and metabolized by Roseburia—this structure-microbiota adaptability further ensures efficient SCFA production.

SCFAs can activate G protein-coupled receptors (GPCRs): among these, FFAR2 (GPR43) and FFAR3 (GPR41) couple with Gi/o proteins after activation, reducing intracellular cAMP levels (138, 139); other GPCRs like GPR109A are involved in inhibiting pro-inflammatory factor release (139). This signaling pathway can not only inhibit the expression of liver pro-inflammatory cytokines (e.g., IL-6, TNF-α), reduce chronic inflammation to block the progression of chronic liver disease (CLD) to HCC (83), but also regulate T cell differentiation (e.g., enhancing Treg function, inhibiting Th17 cells) to optimize the liver immune microenvironment (16).

SCFAs (especially butyrate) act as HDAC inhibitors, which can increase histone acetylation levels, open chromatin structure to activate tumor suppressor gene transcription (140, 141), and promote epigenetic reprogramming of DCs and macrophages to enhance anti-tumor immune responses (142). At the same time, SCFAs induce apoptosis of HCC cells (e.g., HepG2) by inhibiting HDAC activity to activate pro-apoptotic genes (e.g., Bax, p21), and can enhance the activity of NK cells and cytotoxic T cells to inhibit tumor immune escape (141).

The GPCR signal and HDAC inhibition pathway of SCFAs can act synergistically: they jointly reduce liver pro-inflammatory factor levels, inhibit the infiltration of immunosuppressive cells (e.g., MDSCs), reshape the immunosuppressive TME (83); they can also regulate bile acid metabolism via the gut-liver axis to further optimize liver immune homeostasis (142). This TME remodeling effect can improve the efficacy of immune checkpoint inhibitors (e.g., anti-PD-1/PD-L1) on HCC (36). From a clinical application perspective, specific polysaccharides (e.g., ginseng, seaweed polysaccharides) can increase SCFA production via the above pathways, significantly inhibiting the growth of HCC cells (e.g., HepG2) (143, 144); targeted regulation of the gut microbiota-SCFAs axis is expected to become an auxiliary strategy for HCC immunotherapy, such as combined intervention with probiotics or dietary fiber (77, 145).

### 5.4 PD-1/PD-L1 immune checkpoint pathway

The PD-1/PD-L1 pathway is the core target of HCC immunotherapy; polysaccharide-microbiota interaction modulates this pathway by reshaping the immunosuppressive TME and enhancing ICI efficacy, which is the convergence of all above metabolic and inflammatory pathways. The regulatory effects of the above metabolic and inflammatory pathways ultimately converge on the PD-1/PD-L1 immune checkpoint pathway, which is the core target of HCC immunotherapy—polysaccharide-gut microbiota interaction modulates this pathway through multiple dimensions, providing a new strategy to improve the efficacy of immune checkpoint inhibitors. The PD-1/PD-L1 pathway is the core mechanism of HCC immunotherapy: tumor cells can express PD-L1 to bind to PD-1 on immune cell surfaces (e.g., T cells), achieving immune surveillance escape and promoting tumor progression (146–149). Inhibitors like anti-PD-1/PD-L1 antibodies can block this pathway, restore T cell function, and have been applied in HCC treatment (150, 151), but the overall response rate of monotherapy is only ~20%—this is closely related to the TME’s immunosuppressive state and the complexity of PD-L1 expression regulation (152, 153). PD-L1 expression is affected by multiple factors such as gene variation, epigenetic modification, and post-transcriptional regulation (154), and this pathway is associated with pathways like Wnt/β-catenin, which can further promote tumor immune escape and treatment resistance (16); in addition, the gut microbiota (as a key regulatory factor) can affect the HCC immune microenvironment and immune responses via the gut-liver axis, thus potentially regulating the therapeutic effect of the PD-1/PD-L1 pathway (21, 77).

As natural bioactive substances, polysaccharides can directly or indirectly regulate gut microbiota composition and function, thus affecting the body’s overall immune response: mushroom polysaccharides can regulate immune responses by adjusting gut microbiota composition and metabolite activity (125); APS have shown anti-cancer activity in preclinical studies—they can inhibit HCC cell proliferation and regulate the TME, and their mechanism partially depends on indirect gut microbiota regulation (96, 106); specific plant-derived polysaccharides (e.g., those used to intervene in antibiotic-associated diarrhea) can promote the normalization of gut microbiota structure, with effects close to the normal control group (155). The chemical structure of polysaccharides (e.g., glycosidic bond type) determines their interaction mode with the gut microbiota. Products like SCFAs produced by microbial metabolism of polysaccharides can participate in the immunoregulation process (155), and the regulatory effect of polysaccharides on the microbiota can also be mediated via pathways like NF-κB, MAPK, and PI3K-Akt, thus affecting immune cell function (156–159).

The gut microbiota can regulate the HCC immune microenvironment via the gut-liver axis, thus affecting PD-1/PD-L1 pathway activity: the gut microbiota and its metabolites activate innate immune responses, and regulate PD-L1 expression and T cell activity by acting on receptors and transcription factors in signaling pathways such as MAPK, TGF-β and PI3K-Akt (160). These effects realize the regulation of the tumor immune microenvironment (160); microbiota composition differences caused by different HCC etiologies (e.g., metabolic dysfunction-associated liver disease, viral hepatitis) are closely related to tumor immune response levels; the gut microbiota is a key prognostic factor for the therapeutic efficacy of immune checkpoint inhibitors, represented by anti-PD-1 inhibitors. FMT or prebiotic intervention can indirectly affect the PD-1/PD-L1 pathway by regulating microbial balance. Fecal microbiota transplantation regulates the Th1/Th2 immune balance and inhibits specific immune diseases through this pathway. This finding suggests that gut microbiota reshaping can improve the therapeutic efficacy of PD-1/PD-L1 targeted therapy (161, 162); microbiota composition differences in HCC patients are related to the response rate of anti-PD-1 combination therapy, and microbiota dysbiosis may lead to reduced treatment response rates (163, 164); in terms of signal pathway interaction, the gut microbiota can affect PD-L1 expression and tumor immune escape by activating or inhibiting key pathways like Wnt/β-catenin. For example, microbiota dysbiosis in HCC may activate the Wnt/β-catenin pathway, leading to resistance to PD-1/PD-L1 inhibitors; conversely, normalizing microbiota structure can restore immune balance and improve tumor sensitivity to immune checkpoint inhibitors (36).

The interaction between polysaccharides and the gut microbiota can indirectly regulate the PD-1/PD-L1 pathway in multiple ways: polysaccharides are metabolized by gut microbes to produce metabolites such as short-chain fatty acids, including metabolites from prebiotic polysaccharides. These metabolites regulate immune signaling pathways such as NF-κB and MAPK, and further affect the expression of PD-L1 in the tumor microenvironment. This is the indirect regulatory mechanism of the polysaccharide-gut microbiota interaction on the PD-1/PD-L1 pathway. For example, prebiotics can restore immune balance while reshaping the gut microbiota, and produce a synergistic effect with PD-1/PD-L1 immune checkpoint inhibitors to improve HCC treatment efficacy (36); APS may exert anti-tumor effects by directly regulating PD-L1 expression (96); in terms of overall immune regulation, microbiota changes regulated by polysaccharides can affect the functions of immune cells (e.g., macrophages, T cells), thus regulating the PD-1/PD-L1 axis. For example, in HCC, this interaction can reduce PD-L1-mediated immunosuppression by improving the TME (157); specific plant polysaccharides can alleviate immune dysfunction by regulating Th1/Th2 immune balance and the PD-1/PD-L1 pathway (161); in terms of potential therapeutic significance, the polysaccharide-gut microbiota interaction provides a new strategy for HCC immunotherapy. For example, combining prebiotics, polysaccharides with immune checkpoint inhibitors can improve treatment response rates (36); gut microbiota intervention preparations based on polysaccharides may become important non-invasive biomarkers or new therapeutic directions in HCC immunotherapy (163, 164).

## 6 Common mechanisms and etiology-related differential characteristics of polysaccharides in HCC immunotherapy

Polysaccharides exhibit both universal regulatory effects and etiology-specific targeting characteristics in HCC immunotherapy—their common mechanisms lay the foundation for broad-spectrum application, while the differential characteristics are driven by the heterogeneity of TME caused by different HCC etiologies.

### 6.1 Common roles of polysaccharides in HCC immunotherapy

Regardless of HCC etiology, the core role of polysaccharides revolves around reshaping the immunosuppressive TME and enhancing immune treatment responses. Algal polysaccharides can promote the activation of immune cells (e.g., T cells) by inhibiting the activity of immunosuppressive cells (e.g., TAMs, MDSCs), and their targeting of the lactylation process can enhance T cell anti-tumor activity to improve anti-PD-1/PD-L1 sensitivity (165). Algal polysaccharides can also affect angiogenesis and TME components by regulating oxidative stress-related pathways (166). Plant polysaccharides can reprogram the TME by inhibiting angiogenesis and macrophage polarization, enhancing anti-tumor immunity (20). Marine polysaccharides can enhance immune cell function while reducing the off-target toxicity of immunotherapy (125). APS can directly exert anti-cancer effects via pro-apoptosis, anti-proliferation, and anti-epithelial-mesenchymal transition, and indirectly assist immunotherapy (167). In addition, polysaccharides generally target key oncogenic signaling pathways like PI3K/AKT/mTOR, which are involved in tumor progression and the formation of immunosuppressive microenvironments in HCC of different etiologies (168). Polysaccharides can exhibit common therapeutic effects by indirectly blocking pathway activation mediated by the SH3D21 gene (100). With low immunogenicity, good biocompatibility, and tumor-targeting ability, polysaccharides are suitable as drug carriers or immunomodulators (166). Their tumor-targeting ability is a common advantage in treating HCC of various etiologies, and can be used for targeted drug delivery in nanoplatforms (169). Polysaccharides can provide a unified solution for immunotherapy of HCC of various etiologies (170–173).

### 6.2 TME heterogeneity dominates differences in polysaccharide targeting strategies

Although polysaccharides exhibit common regulatory effects in HCC immunotherapy across different etiologies, TME heterogeneity caused by HCC etiology (e.g., metabolic-associated fatty liver disease, viral hepatitis, post-transplant recurrence) is the key reason for differences in polysaccharide therapeutic effects and targeting strategies. Steatohepatitic HCC caused by metabolic dysfunction-associated fatty liver disease (MAFLD/MASH) exhibits unique TME characteristics (e.g., lipid deposition-related immunosuppression, prominent hypoxia) due to fat accumulation and chronic inflammation (174). Such HCC activates oxidative stress pathways like SHP2/PI3K, promoting angiogenesis and invasion (168). The targeted regulation of the lactylation process and oxidative stress pathways by algal polysaccharides is more suitable for this type of HCC (166). The TME of viral hepatitis-associated HCC (HBV/HCV) is rich in virus-specific immune cells (e.g., CTLs). Although APS can exert broad-spectrum effects via pro-apoptosis and anti-proliferation, there are no reports on its specific regulation of virus antigen-related inflammation (166). Post-transplant recurrent HCC has more significant TME immune suppression due to enhanced patient immune tolerance; polysaccharide monotherapy has poor efficacy and requires combination with other immunomodulators, and there are no clear treatment guidelines at present (175). The TME of metabolism-related HCC is more dependent on macrophage polarization, and plant polysaccharides or algal polysaccharides are more likely to show efficacy in regulating this process.

Differences in the activation of key molecular pathways in HCC of different etiologies further lead to specificity in polysaccharide mechanisms of action and treatment responses. The metabolic reprogramming characteristics of metabolism-related HCC make the immunomodulatory mechanism of algal polysaccharides targeting the lactylation process more targeted (166). Although polysaccharides derived from Antrodia cinnamomea have shown anti-tumor activity, no differences in their mechanisms in HCC of different etiologies (e.g., viral, metabolic) have been found (176). Although the PI3K/AKT/mTOR pathway is activated in all HCC types, metabolism-related HCC has more prominent hypoxia, so the inhibitory effect of polysaccharides on this pathway is more significant (168). Viral hepatitis-associated HCC may be dominated by virus-related pathways like NF-κB, and the regulation of these pathways by polysaccharides has not been clarified. The tumor microenvironment of metabolism-related HCC shows obvious immunosuppressive characteristics (177). This microenvironment is more conducive to algal polysaccharides exerting their effect of enhancing the sensitivity of immunotherapy, leading to a better treatment response (177). For pathological subtypes like scirrhous HCC with different TME characteristics, polysaccharide targeting delivery strategies need adjustment (e.g., optimizing nanoplatforms). In terms of prognosis, recurrent HCC in immunosuppressed patients has a poor prognosis, and metabolism-related HCC is often accompanied by an immunosuppressive TME. Polysaccharides as adjuvant therapy are more likely to show efficacy in HCC with low-risk etiologies, but there is no direct clinical data to support this (177).

## 7 Summary

This review systematically clarified the regulatory mechanisms by which the gut microbiota modulates the HCC immune microenvironment via the “gut-liver axis” and four major metabolic axes (bile acids, SCFAs, LPS, tryptophan)—for instance, secondary bile acids inhibit NKT cell recruitment via FXR/TGR5, SCFAs enhance CD8^+^T cell cytotoxicity via GPCR/HDAC, and LPS induces M2 macrophage polarization by activating TLR4/NF-κB; this regulation directly affects HCC progression and the efficacy of ICIs. The study also confirmed that polysaccharides can selectively regulate the gut microbiota based on their structural characteristics (molecular weight, glycosidic bonds, etc.) (e.g., high-molecular-weight polysaccharides promote Bifidobacterium proliferation, while low-molecular-weight polysaccharides are fermented by Bacteroides), and further regulate immune cell functions via substances such as SCFAs and indole derivatives produced by microbial metabolism; meanwhile, polysaccharides bidirectionally regulate core pathways (TLR/NF-κB, bile acid-FXR, PD-1/PD-L1) to reshape the HCC immunosuppressive microenvironment. Additionally, the study verified that nanopolysaccharides (e.g., biotinylated aldehyde alginate-doxorubicin micelles) can target tumors and increase SCFA production by combining with the “gut-liver axis”, and confirmed that TME heterogeneity caused by different HCC etiologies (MAFLD, HBV/HCV, post-transplant recurrence) affects polysaccharide efficacy—for example, algal polysaccharides are more suitable for MAFLD-related HCC.

This review constructed a multi-dimensional regulatory framework for the polysaccharide-gut microbiota-liver immune microenvironment. It integrated the precise regulation of gut microbiota by polysaccharides, metabolite-mediated immune regulation, and the synergistic effects of core signaling pathways, and overcame the limitations of traditional single-target therapy. This study built an association model between polysaccharide structural characteristics, microbiota-specific responses and immune effects, and clarified the differences in target bacteria and metabolite production of polysaccharides with different structures, which provides a structure-function basis for the design of precise polysaccharide preparations. This review combined nanotechnology with the gut-liver axis mechanism to develop a nanopolysaccharide system with tumor-targeted delivery and gut microbiota regulation functions, which enriches the delivery strategies for HCC immunotherapy. It also proposed targeted polysaccharide intervention strategies based on HCC etiological heterogeneity, abandoned the one-size-fits-all treatment model, and provided a new direction for precise immune adjuvant therapy in HCC patients with different etiologies.

## 8 Limitations and challenges

The clinical application of polysaccharides for HCC immunotherapy is hindered by multiple challenges, which are interrelated and restrict the translational potential of this field. The application of polysaccharides in immunotherapy for HCC still faces multiple challenges, which are interrelated and restrict their potential for clinical translation. Firstly, the primary structure and chain conformation of polysaccharides have a crucial impact on their anti-HCC activity. Structural modification can enhance their biological activity, but the inherent structural complexity of natural polysaccharides (such as differences in molecular composition and configuration) makes it difficult to ensure batch-to-batch consistency, affecting the stability of biological activity and the reproducibility of research (178). Secondly, as biological macromolecules, the safe dose window and hepatotoxicity threshold of polysaccharides have not been systematically clarified. In HCC patients with impaired liver function (such as those with cirrhosis), improper dose control may trigger unexpected immune responses through the activation of Toll-like receptor (e.g., TLR2/TLR4) pathways, requiring in-depth verification of their mechanism of action using tools such as monoclonal antibodies. In addition, although polysaccharide-based nano-delivery systems have shown advantages in improving drug targeting, controlled release performance, and reducing systemic toxicity due to their good biocompatibility, modifiability, and inherent liver-targeting ability (e.g., galactose modification) (169), their clinical translation is still limited by bottlenecks such as the morphological uniformity of nano-carriers, *in vivo* stability, and large-scale preparation processes. In the future, focus should be placed on the analysis of the minimum effective structural units, optimization of GMP-grade raw material processes, and design of intelligent responsive carriers to promote the precise application of polysaccharides in HCC immunotherapy.

First, polysaccharides exhibit significant structural heterogeneity and insufficient batch consistency due to differences in molecular weight, glycosidic bonds, and branching degree; this affects the stability of their biological activity, research reproducibility, and the unification of regulatory standards, making it hard to establish structure-function correlations. Although nano-delivery can enhance targeting and stability (179, 180), breakthroughs are still needed via GMP-grade raw material processes, analysis of the minimum effective structural unit (MESU), and nanocarrier targeting optimization. In addition, polysaccharides are easily digested and fermented in the upper gastrointestinal tract, making it difficult to reach the “target bacteria” site in the intestine, resulting in weak targeting and clinical effects; it is necessary to design colon-targeted drug delivery systems, explore prebiotic/probiotic combinations, and adjust fermentation sites according to structural parameters. When combined with immunotherapy, the differences in treatment responses caused by the gut microbiota and the impact of polysaccharides on the TME require first conducting small-scale mechanism verification and embedding microbiome and immune function monitoring in clinical studies.

While the structural heterogeneity of polysaccharides remains unresolved, the unclear causal chain between specific “effector bacteria/metabolites” and immune regulatory nodes further hinders the translational application of this field. For example, the anti-tumor effect of Hua’er polysaccharide (HP) is related to chenodeoxycholic acid (CDCA), but there is a lack of precise attribution of key microbiota and immune pathways; although the bile acid-NKT pathway in HCC provides a causal model (93, 181), it still limits the development of patient stratification and companion diagnostics. At the same time, intratumoral microbes have low biomass and are prone to contamination, so research requires high-sensitivity detection and strict negative controls—otherwise, false attribution is easy; it is necessary to establish aseptic operating specifications, improve negative controls and host background deconvolution methods, and combine spatial omics for *in situ* verification (93).

Furthermore, the gut microbiota of HCC patients is reshaped by cirrhosis, previous antibiotic treatment, and diseases/intervention methods, interfering with the evaluation of immune effects (182), leading to the masking of effective signals in clinical studies; it is necessary to reduce interference via strict inclusion/exclusion criteria, treatment washout periods, stratified correction of intervention factors, and combination with real-world cohorts. At present, there are no unified standards for sampling procedures and biomarker detection in this field, and no standardized sample collection, immune evaluation endpoints, or microbiome transformation frameworks (183), hindering data comparison and regulatory recognition; it is necessary to adopt “microbiota-metabolism-immunity” composite endpoints and follow standardized transformation frameworks. Finally, abnormal activation of the gut microbiota and TLR4 pathway may exacerbate liver inflammation or induce liver damage (184), but the safety threshold and dose window of polysaccharide intervention are unclear; it is necessary to evaluate the immune activation threshold and hepatotoxicity in preclinical studies, and set dose escalation designs and liver function monitoring in clinical trials. Notably, inhibiting Notch1 signaling promotes excessive hepatic inflammation by downregulating the β-catenin/GSK3β pathway, aggravating liver injury; this suggests polysaccharides have a narrow safe dosage window in HCC patients with impaired liver function, and improper dosing increases liver injury risk, hindering clinical translation (185).

To address the potential hepatic injury risk of polysaccharides, future studies need to establish etiology-specific polysaccharide dose standards based on HCC subtypes (e.g., MAFLD-related HCC, HBV-related HCC) and liver function status of patients; meanwhile, combining polysaccharides with TLR4 antagonists (e.g., TAK-242) can block the off-target activation of the TLR4/NF-κB pathway at high polysaccharide doses, thus reducing the risk of liver inflammation while maintaining the anti-tumor effect of polysaccharides.
