# Correction: Polysaccharides-gut microbiota interaction: mechanisms regulating the hepatocellular carcinoma immune microenvironment

**DOI:** 10.3389/fimmu.2026.1923029

**Published:** 2026-07-03

**Authors:** Wei Peng, Kai Xiong, Yuyang Zheng, Jiahan Zheng, Yuanyuan Zhong, Jihao Yang, Yuchuan Jiang

**Affiliations:** 1 Department of Graduate School, The First Clinical Medical College of Gannan Medical University, Gannan Medical University, Ganzhou, China; 2 Department of Gastroenterology, The Second Affiliated Hospital, Jiangxi Medical College, Nanchang University, Nanchang, China; 3 Department of Pediatric Surgery, The First Clinical Medical College of Gannan Medical University, Ganzhou, China; 4 Department of Gastroenterology, Pingxiang People’s Hospital, Pingxiang, Jiangxi, China; 5 School of Acupuncture and Tuina, Guizhou University of Traditional Chinese Medicine, Guiyang, China

**Keywords:** Gut microbiota, gut-liver axis, hepatocellular carcinoma, natural polysaccharides and nanopolysaccharides, tumor immune microenvironment

In the published article, an incorrect **Funding** statement was provided. The **Funding** statement was published as:

“The author(s) declared that financial support was received for this work and/or its publication. This review was supported by the National Natural Science Foundation of China (Grant No. 82560529), and Jiangxi Provincial Natural Science Foundation (Grant No. 20252BAC200580).”

The correct **Funding** statement is:

“The author(s) declared that financial support was not received for this work and/or its publication.”

In the published article, the **Generative AI statement** was incomplete and did not fully reflect the use of AI-assisted tools during manuscript and figure preparation. The statement was published as:

“The author(s) declared that generative AI was not used in the creation of this manuscript.”

A correction has been made to the section **Generative AI statement**:

“Generative AI tools were used only for language polishing, initial mind-map assistance, and visual refinement of figures based on author-designed figure frameworks. The authors subsequently reviewed, edited, and verified all AI-assisted visual and textual content to ensure accuracy and consistency with the manuscript, and take full responsibility for the final content.”

The original version of this article has been updated.

